# Cost of chronic and episodic migraine patients in continuous treatment for two years in a tertiary level headache Centre

**DOI:** 10.1186/s10194-019-1068-y

**Published:** 2019-12-30

**Authors:** Andrea Negro, Paolo Sciattella, Daniele Rossi, Martina Guglielmetti, Paolo Martelletti, Francesco Saverio Mennini

**Affiliations:** 1 Department of Clinical and Molecular Medicine, Sapienza University, Rome, Italy; 2 Regional Referral Headache Centre, Sant’Andrea Hospital, Rome, Italy; 3 Economic Evaluation & HTA (CEIS- EEHTA), Faculty of Economics, University of Rome Tor Vergata, Rome, Italy; 4 Department of Statistical Sciences, Sapienza University of Rome, Rome, Italy; 5 Institute for Leadership & Management in Health, Kingston University London, London, UK

**Keywords:** Cost of illness, Migraine, Chronic migraine, Episodic migraine, Burden of disease, Resource utilization, Cross sectional

## Abstract

**Background:**

Migraine is one of the most common neurological diseases and an estimated 1.04 billion people worldwide have been diagnosed with migraine. Available data suggest that migraine is world widely associated with a high economic burden, but there is great variability in estimated costs that depends on the geographical, methodological and temporal differences between the studies. The purpose of this study was to quantify the annual direct cost of episodic migraine (EM) and chronic migraine (CM), both for the patient and for the National Health System (NHS), using data from subjects who attended an Italian tertiary headache centre. Furthermore, we evaluated comparatively the impact of gender and age on the economic burden of migraine.

**Methods:**

We conducted a retrospective and non-interventional observational analysis of the electronic medical records of subjects with EM and CM who consecutively attended the Regional Referral Headache Centre of Rome and undergoing continuous treatment in the 2 years prior to 31 January 2019. This approach was intended to prevent distorsions due to natural fluctuations in migraine status over time. The collected data included demographic characteristics, number of specialist visits, consumption of medications, diagnostic tests, accesses in the emergency department (ED) and days of hospitalization due to the pathology.

**Results:**

Our sample consisted of 548 patients (85.4% women and 14.6% men): 65.5% had CM and 34.5% had EM. The average annual expenditure per patient was €1482. 82.8% of the total cost (€1227) was covered by the NHS. The main item of expenditure were medications that represented 86.8% (€1286), followed by specialist visits (10.2%), hospitalizations for (1.9%), diagnostic tests for (1%) and ED visits for (0.1%). Costs were significantly higher for women than men (€1517 vs. €1274, *p* = 0.013) and increased with age (*p* = 0.002). The annual direct cost of CM was 4.8-fold higher than that of EM (€2037 vs. €427, *p* = 0.001).

**Conclusion:**

Our results provide a valuable estimate of the annual direct cost of CM and EM patients in the specific setting of a tertiary headache centre and confirm the high economic impact of migraine on both the NHS and patients.

## Introduction

Migraine is one of the most common neurological diseases and it is estimated that around 1.04 billion people worldwide have been diagnosed with migraine [1]. The disease is described as moderate-to-severe headache attacks that can last up to 72 h. The clinical course, at least for patients who attend a headache centre, is characterized by fluctuations from an episodic to a chronic frequency of headache attacks [2]. The third edition of the International Classification of Headache Disorders (ICHD-3) sets the threshold for differentiating episodic migraine (EM) from chronic migraine (CM) at 15 days per month in the last 3 months [3]. Patients with CM often overuse symptomatic drug that may lead to medication overuse headache (MOH) [4]. Almost daily migraine attacks that do not respond to conventional therapies define a refractory CM status [5]. Migraine, in particular CM, is often associated with several comorbidities (e.g. vascular, cardiac, neurological, psychiatric, and pain syndromes), which can complicate therapeutic management [6].

In terms of disability, the Global Burden of Diseases (GBD) has classified migraine as the second world cause of years of life lived with disability (YLDs) [7], and the first cause of YLDs in under 50s in both genders [8]. The full burden and the impact of migraine emerge from the recent results of “My Migraine Voice”, a survey conducted on 11,266 adults with migraine for whom preventive treatments have failed [9]. More than 85% of participants reported that migraine limits their daily activities and negatively affects their professional, private, and social life.

Migraine also has an important economic burden on patients and society. “Cost of illness” is a methodology that allows the economic evaluation of the cost (direct and indirect) caused by illnesses on the population [10]. Direct costs are attributed to medical care for diagnosis, treatment and rehabilitation and include consultations with general practitioner (GP), specialist visits, diagnostic tests, emergency department (ED) visits, hospitalizations, and medications er to treat headaches. Indirect costs include the impact on functional capacity, resulting in reduced social activities, loss of earnings, reduced productivity, loss of education, job losses, unwanted job changes and market replacement for lost domestic services. There is a general agreement that a high percentage of patients never consult a physician for their migraine and will never receive a diagnosis, while only a small percentage regularly consults their physician [11, 12]. As a result, it is yet not possible to precisely quantify the direct costs of migraine [10].

The available data suggest that CM is widely associated with a higher economic burden than EM, but there is a large variability in estimated costs that depends on the geographical, methodological and temporal differences between the studies [13–15]. Differences between countries in the annual cost of care are difficult to interpret because they could also be related to structural differences in healthcare systems, available migraine therapies or differences in awareness and migraine management.

The purpose of this study was to quantify and compare the annual direct cost of EM and CM, both for the patient and for the National Health System (NHS), using the data of subjects attending an Italian tertiary level headache centre. Furthermore, we comparatively evaluated the impact of gender and age on the economic burden of migraine.

## Methods

The study is a cross-sectional cost-of-illness evaluation of the direct cost, carried out on patients with EM and CM who attended the Regional Referral Headache Centre, Department of Clinical and Molecular Medicine, Faculty of Medicine and Psychology, Sapienza University of Rome.

### Data source and participants

The data used for the current economic analysis come from a retrospective and non-interventional observational analysis of the electronic medical records (EMR) of all subjects with EM and CM (assessed with the ICHD-3 [3]) who consecutively attended our tertiary level headache centre and who were in continuous treatment and underwent follow-up visits in the 2 years prior to 31 January 2019 (data collection date). This approach aimed to prevent distorsions due to the natural fluctuations in episodic and chronic status over time [2]. Furthermore, this method allowed a detailed assessment of possible changes in dosage and in the class of pharmacological treatments that occurred during the visits. Prophylactic drugs were prescribed at the daily dosages suggested by the World Health Organization guidelines [16], with the exception of onabotulinumtoxinA which was used as quarterly injections of 195 U based on more recent studies [17]. In our headache clinic, we ask our patients to keep a headache diary to track the characteristics of their headaches, drug use and use of healthcare resource. The EMRs of the patients are updated at each visit with the information obtained from the patient and with the data extrapolated from the headache diary.

Participants were eligible for inclusion in the study if they were ≥ 18 years old and if they had been in continuous treatment for 2 years in order to guarantee the same observation period for each enlisted subject.

### Data on the use of health resources

The data collected included demographic characteristics, number of specialist visits, number of diagnostic tests (echocardiogram, carotid color doppler, brain / cervical magnetic resonance, brain computed tomography, radiographs, blood tests), number of accesses in the emergency department (ED), days of hospitalization due to the pathology and consumption of medications (acute and preventives).

Specialist visits and diagnostic tests are partially funded by the NHS, while ED visits and hospitalizations are entirely financed by the NHS. Depending on the class of drug, symptomatic and prophylactic medications may be or totally funded by the NHS or ma be partially or totally charged to the patient. Acute non-specific migraine medications and nutraceutics are responsibility of the patient. Drug use was quantified by developing an algorithm based on each prescribed drug and the daily dosage (number of tablets or drops) multiplied by the number of treatment days.

### Economic study data

The direct cost estimates were the total of all that had been paid or reimbursed by the NHS plus payments of own pocket money Unit costs were collected from publicly available sources in the calendar year 2019. The Regional Rate Nomenclator for Specialist Outpatient was considered for specialist outpatient services [18] while each ED visit was considered to be €241 [19] and each day of hospitalization with the diagnosis-related group (DRG) 564 “Headache> 17 years” was considered equal to €195 [20]. Medication costs were estimated using the reimbursement price of the Regional Health System for the classes of drugs charged to NHS [21], while the costs of classes of drugs partially charged to the NHS or totally charged to the patient were identified from a private site for health care professionals.

### Data analyses

Demographic and clinical characteristics, number of specialist visits, number of diagnostic tests, number of ED visits, days of hospitalization, and consumption of drugs (reimbursed and not reimbursed by the NHS) were evaluated descriptively. The total annual costs were derived from the direct cost of 2 years of treatment and were summarized based on the average and standard deviation. The presence of statistically significant differences between the groups was assessed by Chi-square test and Fisher exact test, where appropriate, for the proportion, *Student’s T* tests for normal distributions and *Mann-Whitney* tests for non-normal distributions. The significance level was set at 0.05 (always corrected if necessary) for all variables.

## Results

### Participants characteristics

The analyzed sample consisted of 548 patients, 468 (85.4%) women and 80 (14.6%) men, aged between 18 and 84 years and an average of 52 years for both genders. According to the ICHD-III diagnostic criteria, 359 (65.5%) subjects had CM and 189 (34.5%) had EM. CM was more frequent than EM in both men (56.3% and 43.8%, respectively) and women (67.1% and 32.9%, respectively), but not significantly.

### Specialist visits

Subjects enrolled in the study received on average 6 follow-up visits during the 2 years of follow-up, corresponding to a visit every 4 months (Table 1). The average number of specialist visits increased with age (*p* = 0.002) and was higher for CM compared to EM (*p* < 0.0001) while there was no difference by gender (Tables 1 and 2). The average annual cost of specialist visits was €153, covered by the NHS for 39.2% (€60), and was higher for CM than EM (€179 vs. €104, *p* < 0.0001) and increased with age (*p* = 0.006) (Tables 3 and 4).

**Table 1:** Healthcare resource use in 2 years by gender and age

Gender	MEN						WOMEN						*p*-value M vs. W	TOTAL						*p*-value Age
Age	18-34 (*n* = 10)	35-44 (*n* = 9)	45-54 (*n* = 23)	55-64 (*n* = 26)	> 65 (*n* = 12)	Total (*n* = 80)	18-34 (*n* = 47)	35-44 (*n* = 54)	45-54 (*n* = 173)	55-64 (*n* = 129)	> 65 (*n* = 65)	Total (*n* = 468)		18-34 (*n* = 57)	35-44 (*n* = 63)	45-54 (*n* = 196)	55-64 (*n* = 155)	> 65 (*n* = 77)	Total (*n* = 548)	
Specialist visits	3.8	4.8	5.7	6.3	5.4	5.5	5.4	5.8	5.8	6.1	6	5.9	NS	5.1	5.7	5.8	6.2	5.9	5.8	0.002
Diagnostic tests	2 (20)	3 (33.3)	1 (4.3)	5 (19.2)	0 (0)	11 (13.8)	7 (14.9)	4 (7.4)	19 (11)	9 (7)	7 (10.8)	46 (9.8)		9 (15.8)	7 (11.1)	20 (10.2)	14 (9)	7 (9.1)	57 (10.4)	NS
ED visits	1 (10)	0 (0)	0 (0)	1 (3.8)	0 (0)	2 (2.5)	1 (2.1)	0 (0)	2 (1.2)	0 (0)	0 (0)	3 (0.6)		2 (3.5)	0 (0)	2 (1)	1 (0.6)	0 (0)	5 (0.9)	NS
Hospitalizations	0 (0)	2 (22.2)	1 (4.3)	1 (3.8)	0 (0)	4 (5)	2 (4.3)	5 (9.3)	11 (6.4)	3 (2.3)	1 (1.5)	22 (4.7)		2 (3.5)	7 (11.1)	12 (6.1)	4 (2.6)	1 (1.3)	26 (4.7)	0.044

*ED* emergency department

**Table 2:** Healthcare resource use in 2 years by migraine status

Diagnosis	CM (*n* = 359)	EM (*n* = 189)	Total (*n* = 548)	*p*-value
Specialist visits	6.8	3.9	5.8	< 0.0001
Diagnostic tests	24	33	57	< 0.0001
Hospitalizations	24	2	26	0.002

*CM* chronic migraine, *ED* emergency department, *EM* episodic migraine.

**Table 3:** Annual cost of specialist visits, diagnostic tests, hospitalizations and ED visits by gender, age and migraine status

Age	MEN								WOMEN							
	N.	Specialist visits		Diagnostic tests		Hospitalizations	DH visits	Total	N.	Specialist visits		Diagnostic tests		ED visits	Hospitalizations	Total
		NHS	PRI	NHS	PRI	NHS	NHS	NHS + PRI		NHS	PRI	NHS	NHS	PRI	NHS	NHS + PRI
18-34	10	39 €	61 €	5 €	2 €	12 €	0 €	119 €	47	56 €	86 €	19 €	4 €	3 €	21 €	189 €
35-44	9	49 €	76 €	27 €	6 €	0 €	163 €	321 €	54	60 €	93 €	12 €	2 €	0 €	54 €	221 €
45-54	23	59 €	92 €	5 €	1 €	0 €	21 €	179 €	173	59 €	92 €	13 €	3 €	1 €	31 €	199 €
55-64	26	65 €	101 €	23 €	4 €	5 €	19 €	216 €	129	63 €	98 €	7 €	2 €	0 €	19 €	189 €
> 65	12	56 €	87 €	0 €	0 €	0 €	0 €	143 €	65	62 €	95 €	12 €	3 €	0 €	15 €	187 €
**Total**	**80**	**57 €**	**88 €**	**13 €**	**3 €**	**3 €**	**30 €**	**194 €**	**468**	**61 €**	**94 €**	**12 €**	**3 €**	**1 €**	**27 €**	**196 €**

*CM* chronic migraine, *ED* emergency department, *EM* episodic migraine, *NHS* National Health System, *PRI* private cost

Costs are calculated in 2019 Euros

The private contribution for specialist visits and diagnostic tests is calculated separately

**Table 4:** Annual cost of specialist visits, diagnostic tests, hospitalizations and ED visits by gender, age and migraine status

Diagnosis	N.	Specialist visits		Diagnostic tests		ED visits	Hospitalizations	Total
		NHS	PRI	NHS	PRI	NHS	NHS	NHS + PRI
CM	359	70 €	109 €	8 €	2 €	1 €	39 €	229 €
EM	189	41 €	63 €	19 €	4 €	1 €	5 €	132 €
**Total**	**548**	**60 €**	**93 €**	**12 €**	**3 €**	**1 €**	**28 €**	**196 €**

*CM* chronic migraine, *ED* emergency department, *EM* episodic migraine, *NHS* National Health System, *PRI* private cost

Costs are calculated in 2019 Euros

The private contribution for specialist visits and diagnostic tests is calculated separately

### Diagnostic tests

The use of at least one diagnostic test during the 2 years of follow-up involved 57 patients (10.4%), and the most frequent examinations were brain MRI (59% of total examinations performed) and carotid color doppler (11% of the total) (Table 1). The use of diagnostic tests was higher among EM patients (*p* < 0.0001) (Table 2**)** while there were no differences for gender and age (Table 1**).** The diagnostic tests had an average cost per patient of €15, covered by the NHS for 80% (€12). The cost was significantly higher for EM than CM (€23 vs. €10, *p* < 0.0001) while there was no association with gender and age (Tables 3 and 4).

### ED visits and days of hospitalization

In the 2 years of observation, only 5 of 548 patients (0.9%) entered the ED, while 26 patients (4.7%) had at least one hospitalization (Table 1). The small sample of patients who entered the ED did not allow any statistical analysis. The number of days of hospitalization was higher among CM patients (*p* = 0.002) and increased with age (*p* = 0.044) while there were no significant differences for gender (Table 2). The mean annual cost for the NHS relating to the hospital management of patients in terms of days of hospitalization was €28 per patient. The cost was higher for CM than EM (€39 vs. €5, *p* = 0.003) without differences for gender and age (Tables 3 and 4).

### Medications consumption

The use of acute and preventive mediactions and the use of specific drugs classes varied according to gender, age and migraine status (Table 5). Women used more calcium channel blockers (*p* = 0.043) and nutraceutics (*p* = 0.028) while men used more ß-blockers (*p* = 0.003).

**Table 5:** Medications use in 2 years by gender, age and migraine status

		ACUTE					PREVENTIVE							
**Gender**	**N.**	**Triptans**	**NSAIDs**	**Combination analgesics**	**Opioids**	**Simple analgesics**	**Antidepressants**	**β-blockers**	**Angiotensin inhibitors**	**OnabotulinumtoxinA**	**Calcium channel blockers**	**Nutraceutics**	**Anxiolytics**	**Antiepileptics**
Men	80	44 (55)	37 (46.3)	45 (56.3)	2 (2.5)	2 (2.5)	25 (31.3)	15 (18.8)	3 (3.8)	45 (56.3)	44 (55)	43 (53.8)	15 (18.8)	6 (7.5)
Women	468	309 (66)	243 (51.9)	231 (49.4)	3 (0.6)	34 (7.3)	171 (36.5)	49 (10.5)	15 (3.2)	314 (67.1)	312 (66.7)	311 (66.5)	106 (22.6)	74 (15.8)
*p-value*		NS	NS	NS	NS	NS	NS	0.003	NS	NS	0.043	0.028	NS	NS
**Age**	**N.**	**Triptans**	**NSAIDs**	**Combination analgesics**	**Opioids**	**Simple analgesics**	**Antidepressants**	**β-blockers**	**Angiotensin inhibitors**	**OnabotulinumtoxinA**	**Calcium channel blockers**	**Nutraceutics**	**Anxiolytics**	**Antiepileptics**
18-34	57	44 (77.2)	34 (59.6)	35 (61.4)	0 (0)	2 (3.5)	10 (17.5)	1 (1.8)	0 (0)	25 (43.9)	34 (59.6)	43 (75.4)	6 (10.5)	7 (12.3)
35-44	63	50 (79.4)	32 (50.8)	38 (60.3)	2 (3.2)	3 (4.8)	25 (39.7)	4 (6.3)	0 (0)	43 (68.3)	49 (77.8)	44 (69.8)	12 (19)	7 (11.1)
45-54	196	144 (73.5)	97 (49.5)	94 (48)	1 (0.5)	7 (3.6)	70 (35.7)	30 (15.3)	7 (3.6)	125 (63.8)	129 (65.8)	141 (71.9)	42 (21.4)	31 (15.8)
55-64	155	92 (59.4)	79 (51)	70 (45.2)	2 (1.3)	12 (7.7)	53 (34.2)	22 (14.2)	5 (3.2)	113 (72.9)	92 (59.4)	91 (58.7)	42 (27.1)	20 (12.9)
> 65	77	23 (29.9)	38 (49.4)	39 (50.6)	0 (0)	12 (15.6)	38 (49.4)	7 (9.1)	6 (7.8)	53 (68.8)	52 (67.5)	35 (45.5)	19 (24.7)	15 (19.5)
*p-value*		< 0.0001	NS	NS	NS	0.011	0.005	0.016	NS	0.003	NS	< 0.0001	NS	NS
**Diagnosis**	**N.**	**Triptans**	**NSAIDs**	**Combination analgesics**	**Opioids**	**Simple analgesics**	**Antidepressants**	**β-blockers**	**Angiotensin inhibitors**	**OnabotulinumtoxinA**	**Calcium channel blockers**	**Nutraceutics**	**Anxiolytics**	**Antiepileptics**
CM	359	220 (61.3)	207 (57.7)	151 (42.1)	4 (1.1)	30 (8.4)	149 (41.5)	45 (12.5)	15 (4.2)	359 (100)	230 (64.1)	200 (55.7)	107 (29.8)	60 (16.7)
EM	189	133 (70.4)	73 (38.6)	125 (66.1)	1 (0.5)	6 (3.2)	47 (24.9)	19 (10.1)	3 (1.6)	0 (0)	126 (66.7)	154 (81.5)	14 (7.4)	20 (10.6)
*p-value*		0.035	< 0.0001	< 0.0001	NS	0.020	0.000	NS	NS	< 0.0001	NS	< 0.0001	< 0.0001	NS
**Total**	**548**	**353 (64.4)**	**280 (51.1)**	**276 (50.4)**	**5 (0.9)**	**36 (6.6)**	**196 (35.8)**	**64 (11.7)**	**18 (3.3)**	**359 (65.5)**	**356 (65)**	**354 (64.6)**	**121 (22.1)**	**80 (14.6)**

*CM* chronic migraine, *EM* episodic migraine, *NSAIDs* nonsteroidal anti-inflammatory drugs.

Older age was associated with a reduction in the use of triptans (*p* < 0.0001) and an increase in the use of simple analgesics (*p =* 0.011), antidepressants (*p =* 0.005), ß-blockers (*p =* 0.016), onabotulinumtoxinA (*p =* 0.003) and nutraceutics (*p* < 0.0001). As preventive medications, patients with CM used more antidepressants (*p =* 0.000) and anxiolytics (*p <* 0.0001) while EM patients used more nutraceutics (*p <* 0.0001). Among acute medications, CM patients used more NSAIDs (*p <* 0.0001) and simple analgesics (*p* = 0.020) while patients with EM used more triptans (*p* = 0.035) and combination analgesics (*p <* 0.0001). The overall mean annual cost of medications was €1286 per patient, of which the 85.1% accounted for preventive treatments. The mean annual cost of acute medications was €191, covered by NHS for the 87.9%, and was significantly higher for women and CM patients and increased with age (Table 6). The mean annual cost of prevenitve medications was €1095, covered by NHS for the 87.6%, and was significantly higher for women (€1321 vs. €1079 for male, *p* = 0.010) and CM patients (€1808 vs. €294 for EM, (*p <* 0.0001) and increased with age (*p <* 0.0001) (Table 6).

**Table 6:** Annual cost of medications by gender, age and migraine status

**Gender**	**N.**	**Acute** **NHS**	*p*-value	**Acute** **PRI**	*p*-value	**Preventive** **NHS**	*p*-value	**Preventive** **PRI**	*p*-value	**Acute** **NHS + PRI**	*p*-value	**Preventive** **NHS + PRI**	*p*-value
Men	80	131 €	0.007	23 €	NS	819 €	NS	106 €	0.009	154 €	0.010	925 €	0.013
Women	468	174 €		23 €		983 €		141 €		197 €		1124 €	
**Age**	**N.**	**Acute** **NHS**	*p*-value	**Acute** **PRI**	*p*-value	**Preventive** **NHS**	*p*-value	**Preventive** **PRI**	*p*-value	**Acute** **NHS + PRI**	*p*-value	**Preventive** **NHS + PRI**	*p*-value
18-34	57	139 €	< 0.0001	17 €	0.045	625 €	< 0.0001	126 €	0.039	156 €	< 0.0001	751 €	0.003
35-44	63	206 €		39 €		902 €		154 €		245 €		1056 €	
45-54	196	200 €		17 €		948 €		148 €		217 €		1096 €	
55-64	155	163 €		22 €		1094 €		130 €		185 €		1223 €	
> 65	77	84 €		33 €		1008 €		113 €		117 €		1120 €	
**Diagnosis**	**N.**	**Acute** **NHS**	*p*-value	**Acute** **PRI**	*p*-value	**Preventive** **NHS**	*p*-value	**Preventive** **PRI**	*p*-value	**Acute** **NHS + PRI**	*p*-value	**Preventive** **NHS + PRI**	*p*-value
CM	359	191 €	0.001	27 €	NS	1451 €	< 0.0001	139 €	NS	218 €	< 0.0001	1590 €	< 0.0001
EM	189	123 €		16 €		24 €		132 €		139 €		155 €	
**Total**	**548**	**168 €**		**23 €**		**959 €**		**136 €**		**191 €**		**1095 €**	

*CM* chronic migraine, *EM* episodic migraine, *NHS* National Health System, *PRI* private cost.

Costs are calculated in 2019 Euros.

### Analysis of total costs

**Fig. 1:**
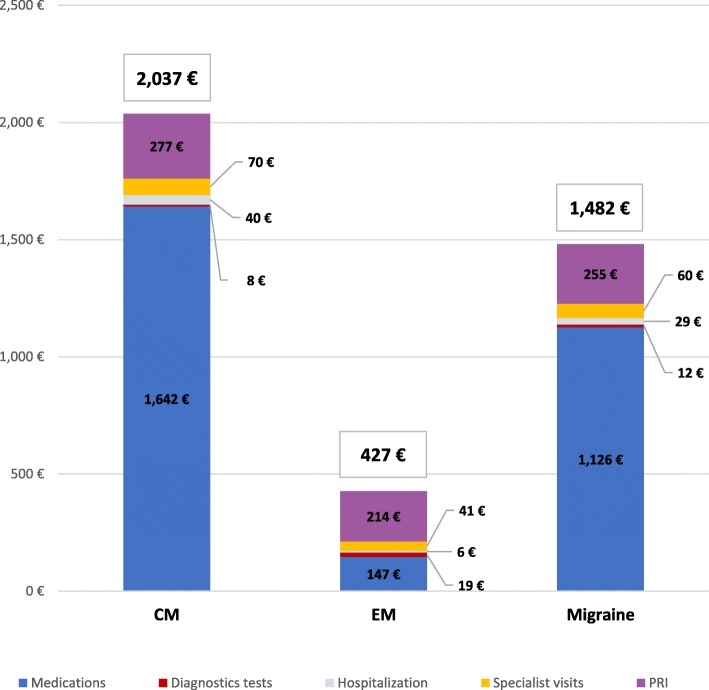
Mean total annual costs per patient by migraine group

The average annual expenditure per patient was €1482 and the variability of expenditure per patient was high, the spending range of was between €51 and €3644, with an inter-quartile difference of €1666 (€511 - €2177) and a coefficient of variation (standard deviation on the arithmetic mean) of the 58.3%. 82.8% of the total cost (€1227) was covered by the NHS. The main item of expenditure was represented by medications that represented 86.8% (€1286), followed by specialist visits for 10.2% (€153), hospitalizations for 1.9% (€28), diagnostic tests for 1% (€15) and ED visits for 0.1% (€1). Costs were significantly higher for women than men (€ 1517 vs. €1274, *p* = 0.013) and increased with age (*p* = 0.002) (Table 7). The annual direct cost of CM was 4.8 times higher than that of EM (€2037 vs. €427, *p* = 0.001) and the difference in average total annual costs between CM and EM was €1610 (Fig. 1).

**Table 7:** Total annual direct cost per patient by gender, age and migraine status

**Gender**	**N.**	**Specialist visits**	**Diagnostic tests**	**ED + Hospitalizations**	**Acute medications**	**Preventive medications**	**PRI**	**Total (±SD)**	*p*-value
Men	80	57 €	13 €	33 €	131 €	819 €	220 €	1274 € (± 1772)	0.013
Women	468	61 €	12 €	28 €	174 €	983 €	260 €	1517 € (± 1685)	
**Age**	**N.**	**Specialist visits**	**Diagnostic tests**	**ED + Hospitalizations**	**Acute medications**	**Preventive medications**	**PRI**	**Total (±SD)**	*p*-value
18-34	57	53 €	17 €	21 €	139 €	625 €	229 €	1085 € (± 1674)	0.002
35-44	63	59 €	14 €	70 €	206 €	902 €	287 €	1537 € (± 1772)	
45-54	196	59 €	12 €	31 €	200 €	948 €	260 €	1510 € (± 1711)	
55-64	155	64 €	10 €	20 €	163 €	1094 €	253 €	1602 € (± 1641)	
>64	77	61 €	11 €	13 €	84 €	1008 €	242 €	1417 € (± 1649)	
**Diagnosis**	**N.**	**Specialist visits**	**Diagnostic tests**	**ED + Hospitalizations**	**Acute medication**s	**Preventive medications**	**PRI**	**Total (±SD)**	*p*-value
CM	359	70 €	8 €	40 €	191 €	1451 €	277 €	2037 € (± 924)	0.001
EM	189	41 €	19 €	6 €	123 €	24 €	215 €	427 € (± 436)	
**Total**	**548**	**60 €**	**12 €**	**29 €**	**168 €**	**959 €**	**255 €**	**1482** € **(± 1705)**	

*CM* chronic migraine, *DH* day hospital, *ED* emergency department, *EM* episodic migraine, *PRI* private cost.

Costs are calculated in 2019 Euros.

The private contribution for specialist visits, diagnostic tests and medications is calculated separately.

## Discussion

Last year, the drug paraphernalia for migraine prophylaxis was enriched with the 3 first-in-class monoclonal antibodies (mAbs) acting on the calcitonin gene-related peptide (CGRP) pathway [22]. The high cost of these drugs requires an adequate selection of patient [23] and probably it will be the same in a few years, when two new classes of migraine-specific drugs (e.g., ditans and gepants) will enter the market [24, 25]. Today more than ever, health systems must face the difficult problem of economic sustainability.

Our study provides a specific quantification of the annual direct cost associated with CM and EM (assessed with the ICHD-3 [3]) based on gender and age of patients in a large population of subjects attending an Italian tertiary level headache centre. The NHS funded 82.8% (€1227) of the annual cost of migraine (€1482) while patients had an annual personal expenditure of €255 as a contribution for specialist visits, diagnostic tests, and medications. The annual cost of CM was €2.037 and that of EM was €427. The cost charged to the NHS was €1760 for CM and €212 for EM, while the cost for the patient was €277 for CM and €215 for EM.

As expected in a headache center, the percentage of patients with CM (65.5%) was significantly higher than that estimated in the general population (2–4%) [26]. The costs for any item of expense were higher for CM expecting the cost of diagnostic tests. The annual cost of prophylaxis was €1095 of which 82% (€902/year per patient) was due to onabotulinumtoxinA and 18% (€193/year per patient) due to oral preventives. The entire sample of patients with CM was treated with onabotulinumtoxinA and this explains how this treatment had an impact on the annual cost estimates. As a result, the number of visits to our headache centre was almost double for CM compared to EM due to the quarterly onabotulinumtoxinA injection program. Patients with CM, compared to those with EM, have used significantly more antidepressants and anxiolytics and this finding is consistent with the greater impact that CM has on quality of life and mood. CM is often associated with a loss of efficacy of triptans and, indeed, the use of triptans was greater among EM patients while CM patients used more NSAIDs and simple analgesics. Furthermore, patients with CM had 12 times more days of hospitalization than patients with EM. As for the other expense items, the highest cost observed in EM in terms of diagnostic examination is surprising only since most of the patients with CM had been in a chronic state for several years and had already undergone a diagnostic investigation to exclude the biological cause before attending our headache centre.

We decided to calculate the annual direct cost by analyzing the patients’ EMR in continuous treatment for 2 years to prevent distortions due to the natural fluctuations in episodic and chronic status over time [2]. Drug therapy can change considerably over the course of a year to adapt to the clinical course of migraine. Following this method it was possible to detect any changes in dosage and class of drugs and, therefore, provide a better estimates of the cost of pharmacological treatments. This is a strength of our study compared to others who have used annualization to adapt the survey data considering a time window of few months. Defining the annual cost of migraine on the basis of a quarter multiplied by four may be almost acceptable for medications use but could overestimate diagnostic tests and hospital visits that are likely to be compressed in the period near to the first admission.

From November 2008 to August 2009, the Eurolight study, a multinational cross-sectional survey, was conducted in eight European countries (Lithuania, Germany, The Netherlands, Luxembourg, Italy, Austria, France and Spain) to estimate the annual direct (medications, outpatient health care, hospitalizations and investigations) and indirect per-person costs (in 2009 Euros) for migraine, tension type headache and MOH (assessed with the ICHD-II [27]) on the base of 8412 self-administered questionnaires [15]. The average total annual cost of migraine per-person in all countries was €1222 of which indirect costs accounted for 93% (€1136). Amongst the direct costs of €86 the main contributory categories were outpatient care (€30), followed by diagnostic tests (€19), acute medications (€16), hospitalization (€16) and preventive medications (€5). However, the main uncertainty in the Eurolight estimates is due to the large variation across countries. Eurolight estimates were logically higher than previous estimates for Austria (€885 vs. €768), Italy (€1034 vs. €706), Lithuania (€297 vs. €152), Luxembourg (€1446 vs. €965), and Netherlands (€1524 vs. €867) [28]. The estimate for Italy was based on a sample size smaller than ours (221 vs. 548 subjects, respectively).

In 2009 a systematic review used the preliminary data provided by the Eurolight group, and therefore different from those subsequently published for the Eurolight study, and estimated in €222 (in 2009 Euros) the total annual (direct + indirect) cost per patient in Italy (in seven other European countries in the range between €111 and €649) [29]. The estimates presented in this report have been converted to real Euro to remove the effect of price differences on the comparison of resource use between countries. The mean cost per subject with migraine in the eight countries (€445) was lower than the estimates considered in 2004 (€590) [30] probably due to methodological differences between Eurolight and previous studies [15]. However, the actual expenses in each individual country is better evaluated with nominal estimates as those used in the Eurolight study, which can be compared directly to other local expenses. Our estimate of annual direct cost per patient (€1482) was significantly higher than that formulated by Eurolight (€86) [15]. This finding could have some possible explanations. First, our estimates were based on the amount of each individual medication consumed and we used the market cost of each unit of these drugs to calculate medical costs. In contrast, in the Eurolight study the estimates were based on an average cost per drug. Furthermore, the cost of prophylactic medication was estimated on the assumption that usage reported was stable over time and costs of recommended daily doses were multiplied by 365 days to estimate annual costs. Secondly, Eurolight was conducted before the introduction of onabotulinumtoxinA that entailed an average annual cost per patient of €902 in our population. Thirdly, Eurolight did not take into account the cost of alternative pharmacological treatments, such as nutraceutics, which had an average annual cost of €82 per patient. Other important differencies between our study and Eurolight are related to the populations studied. First, the Eurolight participants were drawn, depending on the country of enrollment, from the general population or among those who visited GPs or neurologists for any reason. On the contrary, our population was composed of patients who attended a tertiary level headache centre and who, therefore, were presumably more severely affected than those enrolled in Eurolight. Secondly, the different setting (e.g. headache centre vs. general population) can easily explain why our population was composed mostly of patients with CM (65.5%). Furthermore, Eurolight considered the cost of migraine patients as a whole population without differentiating the costs of EM and CM. Although all these differences make it difficuly to compare the results, we can assume that the direct cost in Eurolight was probably underestimated.

In the same year of the Eurolight study, the International Burden of Migraine Study (IBMS) [31] evaluated the direct medical costs of CM and EM (assessed with the ICHD-II [27]) in North America (U.S. and Canada) [13] and Western Europe (Germany, France, Italy, Spain, and UK) [14] using global cross-sectional data collected through a web survey administered from February to April 2009. IBMS investigated the use of healthcare resource occurred in the previous 3 months and multiplying the 3-months average healthcare cost by 4. The direct medical cost were calculated in 2010 U.S. or Canadian dollars for North America, and standardized to 2010 euros for Europe and the UK. CM status was associated with significantly higher use of medical resources and total costs compared to EM in all study countries [13, 14]. CM participants had more provider visits, ED and hospital visits, and diagnostic tests. The mean direct cost of care varied widely in the five European countries suggesting differences in migraine management, organization of NHS and reimbursements. The number of subjects evaluated in each country was in the range 55–57 for the CM group and in the range 644–1404 for the EM group. Overall, the annualized costs of care for EM were highest in Spain followed by the UK, Italy, Germany and France. The costs of CM medical care were highest in the UK followed by Spain and Italy and then France and Germany. The IBMS study showed that in Europe the average direct cost of CM was about three times higher than that of EM (€2427/year and €746/year, respectively) [14]. In particular, costs were 3.6-fold higher in UK (€3718 vs. €866/year), 2.3-fold higher in France (€1579 vs. €486/year), 1.5-fold higher in Germany (€1495 vs. €696/year), 2.5-fold higher in Italy (€2648 vs. €828/year), and 2-fold higher in Spain (€2669 vs. €1092/year). The difference between the average total annual costs between CM and EM was €2852 in UK, €1093 in France, €799 in Germany, €1820 in Italy and €1577 in Spain. Considering the Italian estimates, the annual cost for CM was €2648 and the cost of EM was €828. Our results showed lower costs for both groups (€2037 and €427, respectively; difference CM-EM: €1610) but unlike the IBMS study, we did not evaluate the cost of GPs or other specialist visits, nurse practitioners/physicians, transcutaneous nerve stimulators, occipital nerve block procedures and acupuncture. However, the estimates of IBMS were based on patients with primary headaches that were drawn from a pool of registered panelists who expressed willingness to complete health surveys in general, without reference to headache. This explain why 95.1% of the population was made up of EM patients and only 4.9% of CM. On the contrary, we found a proportion between EM an CM (35.5% and 65%, respectively) which was different from that found in the general population which reflects the greater severity of the patients who turns to a tertiary level headache centre.

Unlike the studies mentioned above, ours had the main purpose of assessing the direct cost in the specific setting of a tertiary level headache centre. Two other studies, both conducted in Italy, evaluated the healthcare costs of patients attending a headache centre. The population investigated in both studies was smaller than ours.

D’Amico et al. [32] evaluated the direct and indirect costs of CM and MOH (assessed with the ICHD-3-beta [33]) at the time of structured withdrawal in a headache centre. The estimates were based on the 3-months evaluation. Based on data from 135 patients, the total annual cost per person was estimated at around €10,370, of which €3495 (34%) due to direct healthcare cost, €515 (5%) due to direct non-medical cost and €6360 (61%) due to indirect cost. Our estimate of direct costs is lower (€1482) and this could depend on the different population studied (CM and EM vs. CM and MOH). However, the authors have not distinguished the costs of individual items within each expense category and this makes it difficult to compare their estimate with ours. The cost of prophylaxis (oral preventives and onabotulinumtoxinA) was estimated at €215/year per patient, but the cost of individual drug classes was not provided. We estimated that the annual cost of prophylaxis was €1095 of which 82% (€902/year per patient) is due to onabotulinumtoxinA and 18% (€193/year per patient) is due to oral preventives. In the same study, the annual cost of non-pharmacological treatments (nutraceuticals and behavioral approaches) was €1867 per patient. The cost of individual items of expenditure is not provided but the high cost was probably due to the contribution of behavioral approaches rather than nutraceuticals, while for the latter our study estimated an annual cost of €83 per patient. Also for the cost of diagnostic procedures (e.g. physician visits, diagnostic tests, hospitalizations), estimated at €1296/year per patient, the contribution of the individual expense items was not provided. In our study the combined cost of those three items of expenditure was much lower (€101/year per patient) and this could be explained by a greater number of hospitalizations among patients with MOH.

Berra et al. conducted another Italian study that addressed annual direct healthcare costs (in 2013 Euros) in a tertiary level headache centre [34]. The costs were estimated using ad hoc questionnaire on medical resource use during the previous 3 months. Based on data from 92 patients (51 with CM and 41 with EM, assessed with the ICHD-3-beta [33]), the mean annual cost was €1480. The annual cost of CM (€2250) was 4.3-fold higher than that of EM (€523). The cost loaded on NHS was €2110 for CM and €468 for EM, while the cost for the patient was €140 for CM and €55 for EM. In our study we considered the same categories of expenses and found almost overlapping results with an average cost of €1482 per year. Also, our estimates of the annual cost of CM (€2037) and EM (€427) were similar to those proposed by Berra et al. For our sample of patients, the cost charged on the NHS was €1760 for CM and €212 for EM, while the cost to the patient was €277 for CM and €215 for EM. The differences between their estimates and ours emerge when we observe the contribution of the individual expense items to the total cost. In their study, the main item of expenditure was hospitalizations that accounted for 53.9% (in ours: 1.9%), followed by medications for 32% (in ours: 86.8%), diagnostic tests for 7.3% (in ours: 1%) and consultations for 6.8% (in ours: 10.2%). These differences may have two main reasons. First, the large variation in the contribution of hospitalizations may depend on a different approach in the management of migraine patients. Second, in our study, medications were the first leading expense and their contribution to the total cost was nearly 3-fold higher than that estimated by Berra et al. More in detail, we found that preventives accounted for 85.1% and acute treatments for 14.9% of the total medications cost while Berra et. found an opposite result with acute treatments accounting for 78.2% and preventives for 21.8%. On the other side, their population was represented by almost the same number of CM and EM patients. In contrast, 65.5% of our patients had CM and were all treated with onabotulinumtoxinA, thus explaining the higher preventives expense.

### Limitations of the study

This study is subjected to a number of limitations and involved has several assumptions. Our analysis covered a large population of 548 patients who attended our tertiary level headache centre continuously for 2 years. Despite this, our estimates cannot be generalized to all Italian headache centres because treatment programs and organizational management vary greatly between different Italian regions.

As regards cost analysis, we calculated only the direct costs strictly related to migraine but not the cost of disorders that are comorbid or secondary to migraine or to its treatment, which have a strong economic impact on migraine management; including these conditions, costs would of course increase even further. Our approach has been designed to capture only the costs starting from the first visit to our centre and related to our migraine management. For this reason, we did not evaluate the costs of the referring GPs (covered by NHS) or of procedures (e.g. transcutaneous nerve stimulators, occipital nerve block procedures and acupuncture) that for different reasons are not used in our centre. Similarly, we did not evaluate the costs of nurse practitioners, physicians and resident doctors, which are entirely covered by the NHS. A further limitation of this study is that we did not calculate indirect costs and the impact of migraine on family life; it would have required a different set-up of the model used for the analysis and it was beyond our aim of specifically calculate the direct costs.

Other limitations include possible selection bias towards more severe migraine participants. Patients attending a third-level headache center often have a history of treatment failures and treatment attempts by general practitioners; indeed, our sample consisted of 65.5% of patients with CM, while the prevalence of CM is much lower. The costs are higher in the CM because patients with a high frequency of attacks require more frequent visits and need more treatments. The selection of more severe patients than those from questionnaire-based surveys based on ICHD criteria can explain why our cost estimates are higher than those found in some previous studies. Therefore, our estimates may not reflect the costs in the general population.

Another important limitation is that the use of healthcare resource has been calculated based on the basis of data from patients’ EMRs and there may be the possibility that patients omitted some information or that doctors have not registered them into the EMR, as there is the possibility that patients have not correctly reported all events in their diary. Health records maintained by national health insurance funds could have provided more reliable data, but they would have created other types of bias considering that the Italian NHS provides universal coverage. However, we believe that the methodology used in our study is a step forward compared to the annualization of data based on self-administered questionnaires for the previous 3–4 months.

## Conclusions

Our results provide a valuable estimate of the annual direct cost of patients with CM and EM in the specific setting of a tertiary level headache centre and confirm the high economic impact of migraine on both the NHS and patients. Patients with CM have had more visits, diagnostic tests and drug use than patients with EM, which led to a direct annual cost of 4.8 times that of EM. Furthermore, costs were significantly higher for women than for men and increased with age.

Cost of illness studies become obsolete due to the change the healthcare systems and the availability of new treatments become available. Governments and decision makers should strongly support these investigations to reveal the true economic and social impact of migraine, particularly when it is chronic.

## Data Availability

Dataset available from the corresponding author on reasonable request.

## Abbreviations

CGRP: Calcitonin gene-related peptide
CM: Chronic migraine
DRG: Diagnosis related Group
ED: Emergency department
EM: Episodic migraine
EMR: Electronic medical records
GBD: Global burden of diseases
ICHD: International classification of headache disorders
mAbs: Monoclonal antibodies
MOH: Medication overuse headache
MRI: Magnetic resonance imaging
NHS: National health system
NSAIDs: Non-steroidal anti-inflammatory drugs
